# Letter from Charles H. Wilcox, M. D.

**Published:** 1861-08

**Authors:** Charles H. Wilcox

**Affiliations:** Camp Kalorama, Georgetown Heights, D. C.


### ART. III.—*Letter from* CHARLES H. WILCOX, M. D.

CAMP KALORAMA, GEORGETOWN HEIGHTS, D. C.,
July 10, 1861.

*My Dear Friend and Colleague:*

I am just in receipt of your letter, for which receive my most sincere thanks. Allow me through you to congratulate the profession in prospect of a Medical Journal in Buffalo. Much will thereby be done for the general good, since in no other place can more valuable material be contributed, certainly not with yourself to collect and provide it. I have had opportunity to judge of the capacity and the activity of the profession since I took to military life, and though my experience is short, I can yet see the want of that discipline one obtains by making diagnosis, and then submitting to the test of a post mortem examination, as has been the case with those I have been accustomed to associate with in Buffalo. The Regiment has been vaccinated. Whatever an analysis can furnish of interest I cannot tell. A report shall be furnished our Association such as is made to the Surgeon General. Practice in camp can never be scientific. I have often asked why it was not, but could never until I had a little experience answer the question. It is this: At Surgeon's call, which is announced in general order, at dress parade, and may vary according to circumstances, but usually in the morning; at that time all the sick who can walk come to the Assistant Surgeon's quarters, and are prescribed for, and here most of the prescriptions are dispensed. In passing them in review it requires aptitude, concentration and quickness of thought, for every one who reports himself to his orderly is marched up under his command. The real sick are soon prescribed for, but the other class is much more difficult and troublesome. Imaginary and feigned sickness, to get excused from duty, gives both surgeon and officers a great deal of trouble.

Our regiment was pieced up wholesale; the inspection at Elmira a mere farce, and many a man passed who never should have been. I received order on arrival here to re-inspect, which I have done, and mustered out about twenty-five men. They will be returned to their homes as soon as papers can be made out and they draw their pay.

I see Dr. Coventry and Dr. Steele quite often; they are well. Prof. Hamilton is also here, dressed in military. We shall move into Virginia in about ten days. What I see published in the papers, as extracts from letters from the camp, is mostly false—done to gratify some one's vanity or ambition—well enough if only true. I send this franked by Mr. Spaulding, who visited our camp last week. Let me hear from you often, and believe me, yours ever,

CHARLES H. WILCOX.

---

THE general practice of the French surgeons in the Crimea was to extract foreign bodies from wounds at an early period, whenever they were easily accessible. The most efficient styptics in arresting hemorrhage, where the blood-vessels could not be conveniently tied, were the perchloride and the persulphate of iron. Amputations were generally resorted to in severe injuries of the limbs, and the results were more favorable than when conservative surgery was attempted. Primary amputations were much more successful than secondary.

